# The use of renin angiotensin aldosterone system inhibitors may be associated with decreased mortality after cancer surgery

**DOI:** 10.1038/s41598-022-10759-y

**Published:** 2022-04-27

**Authors:** Ah Ran Oh, Jungchan Park, Jong-Hwan Lee, Jeong Jin Min, Joonhee Gook, Jae Ni Jang, Seung-Hwa Lee, Kyunga Kim, Joonghyun Ahn

**Affiliations:** 1 Department of Anesthesiology and Pain Medicine, Samsung Medical Center, Sungkyunkwan University School of Medicine, 81 Irwon-ro, Gangnam-gu, Seoul, 06351 Korea; 2 Rehabilitation and Prevention Center, Heart Vascular Stroke Institute, Samsung Medical Center, Sungkyunkwan University School of Medicine, Seoul, Korea; 3 Statistics and Data Center, Research Institute for Future Medicine, Samsung Medical Center, Seoul, Korea; 4 Department of Digital Health, SAIHST, Sungkyunkwan University, Seoul, Korea

**Keywords:** Surgical oncology, Cancer

## Abstract

Renin–angiotensin–aldosterone system (RAAS) inhibitors are antihypertensive agents with conflicting results on protective effects against some types of cancer. In light of these controversies, we aimed to study the effects of RAAS inhibitors in patients undergoing cancer surgery. From March 2010 to December 2019, consecutive adult patients with antihypertensive drug prescription at discharge after cancer surgery were enrolled and divided into two groups according to RAAS inhibitors prescription. The primary outcome was 5-year mortality after surgery. Secondary outcomes included mortalities during 3-year and 1-year follow-ups and cancer-specific mortality and recurrence rates during 5-, 3-, and 1-year follow-ups. A total of 19,765 patients were divided into two groups according to RAAS inhibitor prescription at discharge: 8,374 (42.4%) patients in the no RAAS inhibitor group and 11,391 (57.6%) patients in the RAAS inhibitor group. In 5022 pairs of propensity-score matched population, 5-year mortality was significantly lower in the RAAS inhibitor group (11.4% vs. 7.4%, hazard ratio [HR] 0.73, 95% confidence interval [CI] 0.64–0.83, *P* < 0.001), and 5-year recurrence rate was also lower for the RAAS inhibitor group (5.3% vs. 3.7%, HR 0.82, 95% CI 0.68–0.99, *P* = 0.04). In our analysis, RAAS inhibitor was associated with decreased 5-year mortality in hypertensive patients who underwent cancer surgery. Prescription of RAAS inhibitor in accordance with current guidelines may be associated with improved mortality after cancer surgery.

## Introduction

Renin–angiotensin–aldosterone system (RAAS) inhibitor is recommended as an antihypertensive agent in patients with comorbidities including heart failure, coronary artery disease, diabetes, or chronic kidney disease^1^. Interestingly, many animal studies have suggested that RAAS inhibition could play an important role in preventing cancer initiation and progression by affecting cell proliferation, angiogenesis, and inflammation^2,3^. However, results from population-based studies are controversial^4–8^.

Surgical resection is an essential modality in cancer treatment. In 2015, over 80% of newly diagnosed cancer cases required surgery, and by 2030, 45 million surgical procedures will be annually needed to treat cancers worldwide^9^. Besides, it has been reported that hypertension was present in 37% of cancer patients^10,11^, and the incidence of hypertension in cancer patients even increases up to 80% after chemotherapy^12,13^. Considering these enormous numbers of possible patients who need to be treated for hypertension after cancer surgery, more data seem to be required for evaluating the effect of RAAS inhibitors prescribed postoperatively in cancer surgery.

Therefore, in the present study, adult patients discharged with anti-hypertensive agent prescription after cancer surgery were enrolled and divided into those prescribed RAAS inhibitors and those prescribed the other anti-hypertensive agents. We compared the 5-, 3-, and 1-year mortalities and cancer recurrence rates between those two groups of patients.

## Methods

This study was a retrospective cohort study using a large single-center data from the Samsung Medical Center Cancer Surgery (SMC-CanSur) registry. Approval for this study and the need for individual written informed consent were waived by the Institutional Review Board at Samsung Medical Center (SMC 2020-04-027), because the entire dataset was initially extracted in de-identified form. The cohort for this study was registered at https://cris.nih.go.kr (KCT0005000). This study was conducted in compliance with the declaration of Helsinki and was reported according to the Strengthening the Reporting of Observational studies in Epidemiology guideline.

### Data curation and study population

The data in SMC-CanSur registry were extracted using the “Clinical Data Warehouse Darwin-C” of Samsung Medical Center. It is an electronic system built for investigators to search and compile anonymized medical records from the institutional electronic archive system. Our archive system contains records of more than 2.2 million surgeries, one billion laboratory findings, 100 million disease codes, and 200 million prescriptions. For cancer patients, all medical information related to cancer such as diagnosis date, cancer stage, cancer treatment, metastasis, and recurrence in this system are separately organized and updated as cancer patient data. Mortality data in this system are continuously verified with the National Population Registry of the Korea National Statistical Office using identification number of each patient for mortality statistics at institutions other than ours.

The SMC-CanSur registry is a de-identified cohort consisting of 87,621 consecutive adult patients who underwent cancer surgery from March 2010 to December 2019 at Samsung Medical Center, Seoul, Korea. Cancer surgery is defined as surgical removal of a solid tumor and adjacent tissue at various sites including the brain, neck, breast, thorax, abdomen, colon, and pelvis^14^. For this study, patients without prescription of antihypertensive agents at discharge and patients with in-hospital death were excluded from this registry. The remaining 19,765 patients were divided into two groups depending on whether they were prescribed with RAAS inhibitors at discharge after cancer surgery. RAAS inhibitors were either angiotensin-converting enzyme (ACE) inhibitor or angiotensin II receptor blocker (ARB). Other antihypertensive drugs included beta blocker, calcium channel blocker, and diuretics.

### Study endpoints

The primary study endpoint was post-discharge 5-year mortality. Secondary endpoints included 3-year and 1-year mortalities and cancer-specific mortality and recurrence rates within 5, 3, and 1 years after discharge. To identify cancer-specific mortality, we used causes of death from the National Statistical Office in South Korea which was organized according to International Statistical Classification of Diseases and Related Health Problems codes. Cancer-specific mortality was defined as death due to the first primary cancer diagnosed or a diagnosed cancer other than the first primary cancer^27^. We applied death classification algorithm from Surveillance, Epidemiology, and End Results data which was extensively validated to improve accuracy of underlying cause of death^28^.

### Statistical analysis

Baseline characteristics are presented as mean ± standard deviation (SD) or medians with interquartile range (IQRs) for continuous variables, and numbers with percentages for categorical variables. The differences were compared with the t-test or the Mann–Whitney test for continuous variables and χ^2^ or Fisher’s exact test for categorical variables. Outcomes were compared using Cox regression model in entire and matched population. Hazard ratios (HR) with 95% confidence interval (CI) was reported. In entire population, covariates with a p-value less than 0.2 or clinical relevance were selected for the multivariable analysis. Following variables were retained in the multivariable Cox regression model; age, sex, diabetes, coronary artery disease, heart failure, stroke, deep vein thrombosis, chronic kidney disease, chronic lung disease, anemia, preoperative chemotherapy, preoperative radiotherapy, preoperative metastasis, the number of intraoperatively transfused red bold cell units, continuous infusion of inotropes, duration of operation and preoperative use of RAAS inhibitors. To further reduce selection bias and confounding variables between the two groups, we used propensity-score matching method to generate the groups with well-balanced covariates. Multivariable logistic regression was used to derive propensity score model with all covariates and used the model to estimate the propensity score for each patient as the probability of being included in RAAS inhibitor group. The 1:1 matching using the nearest-neighbor matching method was performed without replacement. To be specific, we matched a patient in the no RAAS inhibitor group with a patient in the RAAS inhibitor group having the nearest propensity score and was selected if the caliper was within 1.5 of the standard deviation of the propensity score logit. The optimal caliper was set in order to achieve an appropriate balance between the groups while maximizing statistical power^26^. An appropriate balance between the groups with an absolute standardized difference (ASD) < 10% suggested successful propensity-score matching. Kaplan–Meier survival curves were constructed and compared with the log-rank test using propensity score matched populations. We estimated the probability of dying of a cancer cause accounting for the competing risk as non-cancer cause death. In addition, we performed subgroup analysis on the association between RAAS inhibitor and 5-year mortality for each cancer types, and the results were presented in forest plot. For sensitivity analysis in the matched population, we estimated the potential impact of unmeasured confounders with an assumed prevalence of 40% on the observed association. This analysis estimates the impact of an unmeasured binary confounder on the measured causal association between a binary exposure and a binary outcome^15^. Statistical analyses were performed with R version 4.0.2 (R Foundation for Statistical Computing, Vienna, Austria). All tests were two-tailed, and a p-value less than 0.05 was considered statistically significant.

## Results

### Patient characteristics

From the SMC-CanSur registry, we excluded 318 patients with in-hospital death and 67,538 patients without prescription of any antihypertensive agent at discharge. The remaining 19,765 patients were divided into two groups according to the prescription of RAAS inhibitors at discharge: 8374 (42.4%) patients in the no RAAS inhibitor group and 11,391 (57.6%) patients in the RAAS inhibitor group. The baseline characteristics of the two groups are summarized in Table 1. The RAAS inhibitor group had more males, higher incidences of anemia, diabetes mellitus, heart failure, and stroke but lower incidences of chronic lung disease, preoperative chemotherapy, radiotherapy, and metastasis. The use of intraoperative inotropes and duration of operation was also lower in the RAAS inhibitor group. The use of other antihypertensive drugs including RAAS inhibitor before and after cancer surgery is presented in Supplementary Table S1. The median duration of RAAS inhibitor use after surgery was 452 (325–568) days in entire population and 378 (253–526) days in matched population. The types of cancer surgery according to the study group are presented in Supplementary Table S2.

**Table 1:** Baseline characteristics.

	Entire population				Propensity-score matched population		
	No RAAS inhibitor	RAAS inhibitor	p-value	ASD	No RAAS inhibitor	RAAS inhibitor	ASD
	(n = 8374)	(n = 11,391)			(n = 5022)	(n = 5022)	
Median follow-up period from discharge, days	1020 (415–1818)	929 (390–1800)					
ARB^a^		10,974 (96.3)				4889 (97.4)	
ACEi^a^		442 (3.9)				138 (2.7)	
**Preoperative medication**							
RAAS inhibitor	485 (5.8)	6854 (60.2)	< 0.001	141.8	485 (9.7)	485 (9.7)	< 0.1
ARB	391 (4.7)	6545 (57.5)	< 0.001	138.9	391 (7.8)	462 (9.2)	5.1
ACEi	97 (1.2)	360 (3.2)	< 0.001	13.8	97 (1.9)	29 (0.6)	12.2
Age, years	64.1 (± 10.5)	63.8 (± 9.9)	0.08	2.5	63.6 (± 10.8)	63.3 (± 9.8)	3.4
Male	4742 (56.6)	6742 (59.2)	< 0.001	5.2	2986 (59.5)	3047 (60.7)	2.5
Current smoking	3257 (38.9)	4455 (39.1)	0.77	0.4	2045 (40.7)	2033 (40.5)	0.5
Diabetes	2638 (31.5)	4757 (41.8)	< 0.001	21.4	1978 (39.4)	2195 (43.7)	8.8
Preoperative metastasis	300 (3.6)	333 (2.9)	0.01	3.7	156 (3.1)	144 (2.9)	1.4
Coronary artery disease	765 (9.1)	1061 (9.3)	0.69	0.6	492 (9.8)	355 (7.1)	9.8
Heart failure	33 (0.4)	72 (0.6)	0.03	3.3	16 (0.3)	10 (0.2)	2.4
Stroke	413 (4.9)	643 (5.6)	0.03	3.2	229 (4.6)	248 (4.9)	1.8
Deep vein thrombosis	26 (0.3)	19 (0.2)	0.05	2.9	13 (0.3)	6 (0.1)	3.2
Peripheral arterial occlusive disease	26 (0.3)	36 (0.3)	1.00	0.1	18 (0.4)	13 (0.3)	1.8
Chronic kidney disease	221 (2.6)	309 (2.7)	0.79	0.5	158 (3.1)	115 (2.3)	5.3
Chronic lung disease	417 (5.0)	487 (4.3)	0.02	3.4	247 (4.9)	196 (3.9)	4.9
Dementia	22 (0.3)	31 (0.3)	1.00	0.2	13 (0.3)	11 (0.2)	0.8
Chronic liver disease	507 (6.1)	645 (5.7)	0.26	1.7	292 (5.8)	252 (5.0)	3.5
Preoperative anemia	2170 (25.9)	3198 (28.1)	0.001	4.9	1314 (26.2)	1355 (27.0)	1.8
**Preoperative care**							
Chemotherapy	427 (5.1)	406 (3.6)	< 0.001	7.5	213 (4.2)	181 (3.6)	3.3
Radiation therapy	326 (3.9)	301 (2.6)	< 0.001	7.0	169 (3.4)	136 (2.7)	3.8
Hormone therapy	22 (0.3)	26 (0.2)	0.73	0.7	14 (0.3)	14 (0.3)	< 0.1
Intensive care unit	20 (0.2)	24 (0.2)	0.79	0.6	13 (0.3)	12 (0.2)	0.4
Continuous renal replacement therapy	1.0 (0.0)	0.0 (0.0)	0.88	1.5	1.0 (0.0)	0.0 (0.0)	2.0
**Operative variables**							
Operation duration, minutes	183.6 (± 97.3)	178.1 (± 94.1)	< 0.001	5.8	177.9 (± 93.8)	175.5 (± 92.0)	2.5
General anesthesia	8349 (99.7)	11,356 (99.7)	1.00	0.2	5011 (99.8)	5009 (99.7)	0.8
Total intravenous anesthesia	1365 (16.3)	1839 (16.1)	0.78	0.4	819 (16.3)	752 (15.0)	3.7
RBC transfusion	1805 (21.6)	2558 (22.5)	0.14	2.2	1075 (21.4)	1170 (23.3)	4.5
Continuous infusion of inotropes	6435 (76.8)	8538 (75.0)	0.002	4.4	3669 (73.1)	3451 (68.7)	9.6

Data are presented as n (%) or mean (± standard deviation). ASD less than 0.1 was deemed to suggest a successful balance between the two groups.

*RAAS* renin–angiotensin–aldosterone system, *ARB* angiotensin II receptor blockers, *ACEi* angiotensin-converting-enzyme inhibitors, *ASD* absolute standardized mean difference, *RBC* red blood cell.

^a^These variables were not retained in the propensity-score matching.

### Clinical outcomes

The median follow-up durations for 5-year mortality from the day of discharge were 1020 (415–1818) days for the no RAAS inhibitor group and 929 (390–1800) days for the RAAS inhibitor group. The RAAS inhibitor group had significantly lower 5-year mortality (11.8% vs. 8.8%, HR 0.75, 95% CI 0.67–0.84, p < 0.001). The 3-year and 1-year mortalities were also lower for the RAAS inhibitor group (9.4% vs. 7.1%, HR 0.74, 95% CI 0.66–0.84, p < 0.001 for 3-year mortality and 4.0% vs. 2.8%, HR 0.72, 95% CI 0.60–0.87, p < 0.001 for 1-year mortality). For the recurrence rate, it was lower for the RAAS inhibitor group during 5- and 3-year follow-ups (5.7% vs. 4.2%, HR 0.83, 95% CI 0.70–0.97, p = 0.02 for 5-year recurrence and 4.0% vs. 2.8%, HR 0.78, 95% CI 0.65–0.95, p = 0.01 for 3-year recurrence), but 1-year recurrence rate was not significantly different between the two groups. Cancer-specific mortalities were also significantly lower in the RAAS inhibitor group regardless of follow-up periods (5.6% vs. 3.9%, HR 0.69, 95% CI 0.58–0.81, p < 0.001 for 5-year mortality, 4.9% vs. 3.5%, HR 0.69, 95% CI 0.58–0.82, p < 0.001 for 3-year mortality, and 2.3% vs 1.6%, HR 0.70, 95% CI 0.55–0.90, p < 0.001 for 1-year mortality) (Table 2).

**Table 2:** Mortalities according to RAAS inhibitor use.

	No RAAS inhibitor	RAAS inhibitor	Unadjusted HR (CI)	p-value	Adjusted HR (CI)	p-value
***Entire population***	n = 8374	n = 11,391				
**Mortality**						
5-year mortality, no (%)	984 (11.8)	1006 (8.8)	0.78 (0.72–0.85)	< 0.001	0.75 (0.67–0.84)	< 0.001
3-year mortality, no (%)	788 (9.4)	805 (7.1)	0.77 (0.70–0.85)	< 0.001	0.74 (0.66–0.84)	< 0.001
1-year mortality, no (%)	334 (4.0)	323 (2.8)	0.71 (0.61–0.83)	< 0.001	0.72 (0.60–0.87)	< 0.001
**Recurrence**						
5-year recurrence, no (%)	479 (5.7)	478 (4.2)	0.77 (0.68–0.88)	< 0.001	0.83 (0.70–0.97)	0.02
3-year recurrence, no (%)	332 (4.0)	316 (2.8)	0.73 (0.62–0.85)	< 0.001	0.78 (0.65–0.95)	0.01
1-year recurrence, no (%)	82 (1.0)	68 (0.6)	0.61 (0.45–0.85)	0.003	0.83 (0.57–1.21)	0.33
**Cancer-specific mortality**						
5-year mortality, no (%)	469 (5.6)	447 (3.9)	0.72 (0.64–0.82)	< 0.001	0.69 (0.58–0.81)	< 0.001
3-year mortality, no (%)	408 (4.9)	393 (3.5)	0.73 (0.63–0.83)	< 0.001	0.69 (0.58–0.82)	< 0.001
1-year mortality, no (%)	192 (2.3)	182 (1.6)	0.70 (0.57–0.86)	< 0.001	0.70 (0.55–0.90)	< 0.001
***Propensity-score matched population***	n = 5022	n = 5022				
**Mortality**						
5-year mortality, no (%)	574 (11.4)	373 (7.4)			0.73 (0.64–0.83)	< 0.001
3-year mortality, no (%)	458 (9.1)	308 (6.1)			0.73 (0.63–0.84)	< 0.001
1-year mortality, no (%)	189 (3.8)	126 (2.5)			0.68 (0.54–0.85)	0.001
**Recurrence**						
5-year recurrence, no (%)	267 (5.3)	187 (3.7)			0.82 (0.68–0.99)	0.04
3-year recurrence, no (%)	182 (3.6)	130 (2.6)			0.79 (0.63–1.00)	0.045
1-year recurrence, no (%)	33 (0.7)	33 (0.7)			1.03 (0.63–1.67)	0.91
**Cancer-specific mortality**						
5-year mortality, no (%)	268 (5.3)	167 (3.3)			0.69 (0.56–0.83)	< 0.001
3-year mortality, no (%)	230 (4.6)	150 (3.0)			0.70 (0.57–0.86)	< 0.001
1-year mortality, no (%)	100 (2.0)	74 (1.5)			0.75 (0.56–1.02)	0.06

Multivariable adjustment included age, sex, diabetes, coronary artery disease, heart failure, stroke, deep vein thrombosis, chronic kidney disease, chronic lung disease, anemia, preoperative chemotherapy, preoperative radiotherapy, preoperative metastasis, the number of intraoperatively transfused red bold cell units, continuous infusion of inotropes, duration of operation and preoperative use of RAAS inhibitors.

*RAAS* renin–angiotensin–aldosterone system, *HR* hazard ratio, *CI* confidence interval.

**Figure 1:**
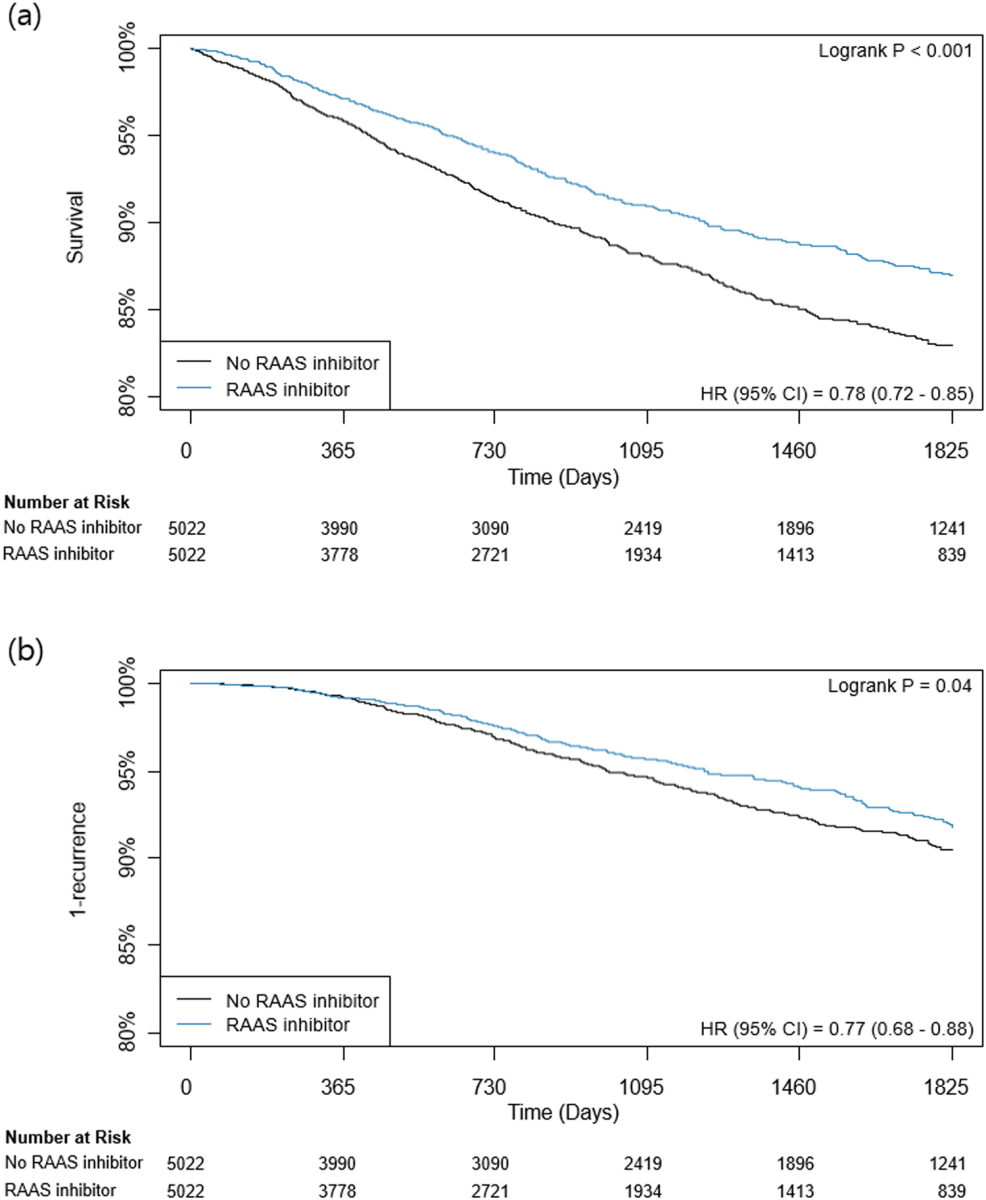
Kaplan–Meier Curves of (**a**) mortality and (**b**) recurrence according to use of RAAS inhibitors during 5 years after cancer surgery. *RAAS* renin–angiotensin–aldosterone system, *HR* hazard ratio, *CI* confidence interval.

**Figure 2:**
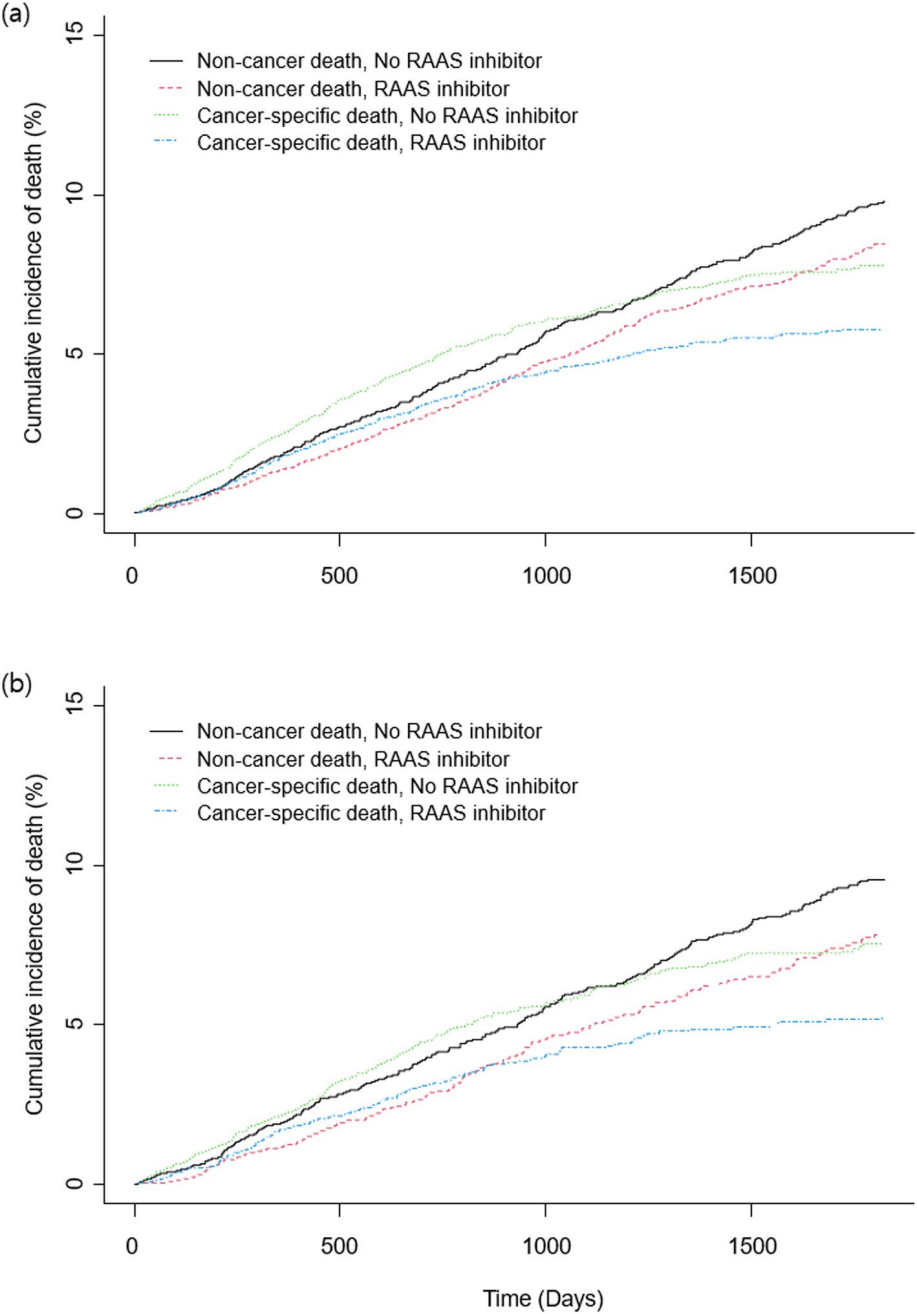
Cumulative incidence of non-cancer and cancer-specific death during 5-years in (**a**) entire and (**b**) matched population. *RAAS* renin–angiotensin–aldosterone system.

After propensity-score matching, 5022 pairs of well-balanced data set with ASD < 10% were generated, and the previous trend persisted in mortality and recurrence rate. The RAAS inhibitor group showed significantly lower mortality during 5-year follow-up (11.4% vs. 7.4%, HR 0.73, 95% CI 0.64–0.83, p < 0.001). (Table 2; Fig. 1). The 5-year cumulative incidence of non-cancer and cancer-specific death is shown in Fig. 2. Cancer-specific death was lower for the RAAS inhibitor group, and the difference of mortality according to RAAS inhibitor use tended to become more marked for cancer-specific death. The sensitivity analysis showed the association between RAAS inhibitor and 5-year mortality after cancer surgery was significant under most circumstances, but the HRs may become statistically non-significant or close to null if the unmeasured confounder has higher-magnitude positive association with the outcome and higher-magnitude inverse association with the exposure of interest (Supplementary Table S3).

**Figure 3:**
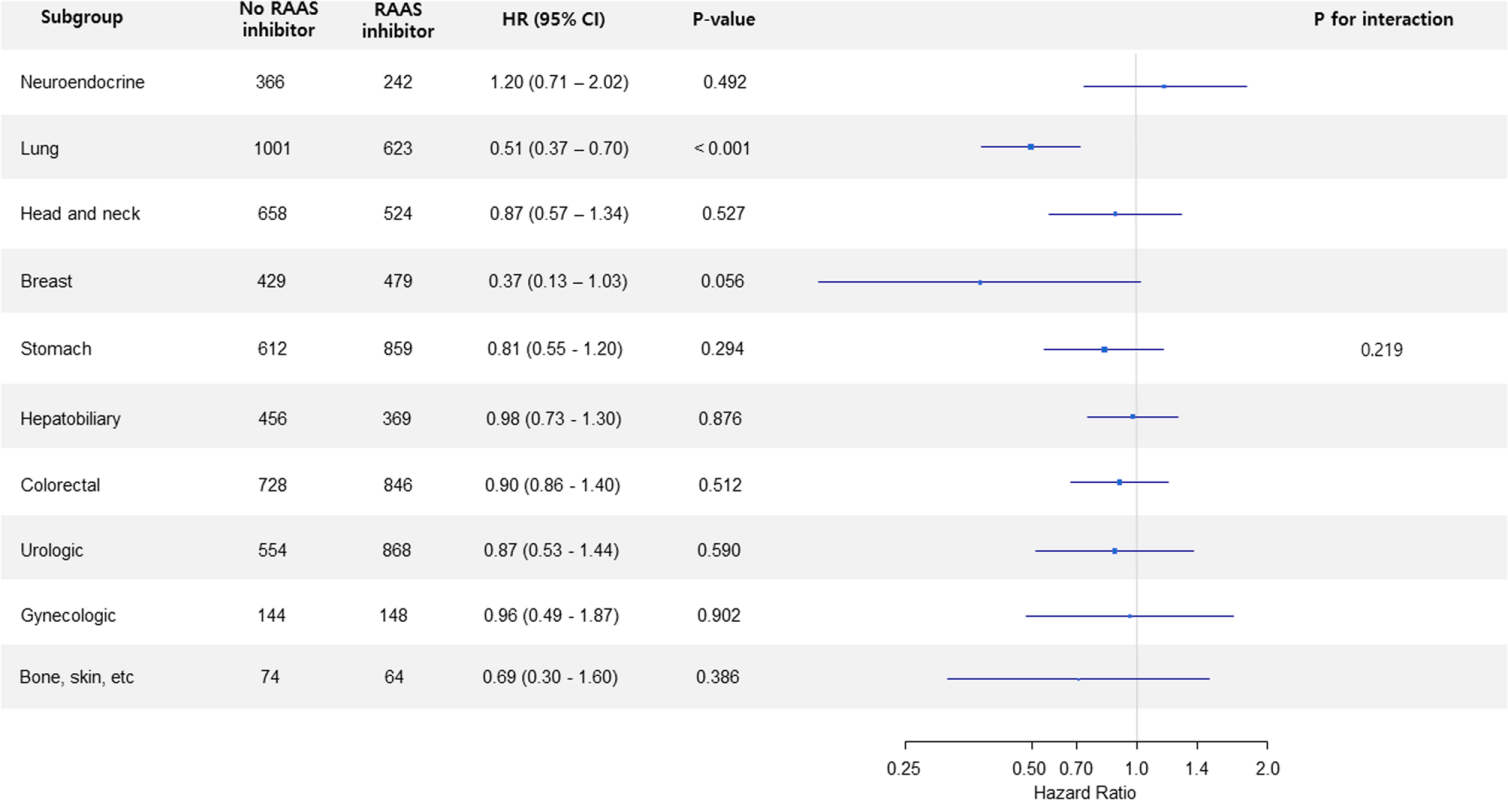
Subgroup analysis for 5-year mortality according to cancer sites. *RAAS* renin–angiotensin–aldosterone system, *HR* hazard ratio.

In subgroup analysis according to cancer site, 5-year mortality was significantly decreased in the RAAS inhibitor group for lung cancer (HR 0.51, 95% CI 0.37–0.70, p < 0.001). The cancer site was not significantly interacted with the association between RAAS inhibitor and mortality. However, there was a heterogeneity according to cancer site on the direction and magnitude of this association, and some of cancers may have not achieved statistical significance due to low power (Fig. 3).

## Discussion

The present analysis showed that RAAS inhibitors' postoperative use was associated with decreased mortality and recurrence rate after cancer surgery except for the 1-year recurrence rate. However, in subgroup analysis, the RAAS inhibitor group showed decreased 5-year mortality only after lung cancer surgery.

To explain this favorable effect of RAAS inhibitor in patients who underwent cancer surgery, we focused on the association with tumor growth and RAAS and the RAAS inhibitors' end-organ protective effects simultaneously. The RAAS plays a vital role in cell growth, maintaining blood pressure, and stabilizing the cardiovascular system's microenvironment^16^. Moreover, ACE and the activation of angiotensin II type 1 receptor (AT1R), critical factors in RAAS, have been associated with tumor growth by stimulating cell proliferation and neovascularization^17–19^. In contrast to AT1R, angiotensin II type 2 receptor (AT2R) activation shows anti-proliferative effects^2,20,21^. Although the exact mechanism for the protective effect of RAAS inhibitor against cancer is not fully known, ACE inhibitors reduce the conversion of angiotensin I to angiotensin II, and ARBs selectively inhibit the unfavorable actions of AT1R by maintaining the protective function of AT2R signaling. In this study, recurrence rate was decreased in the RAAS group, and the cumulative incidence of cacancer-specific death by accounting for competing risk was also lower for the the RAAS group. These findings support that the anti-tumor effect of RAAS inhibitor may be related to our result.

RAAS inhibitors have suggested to provide better end-organ protection such as kidneys, blood vessels, and heart compared with the other antihypertensive agents^22^. Therefore, RAAS inhibitors are highly recommended as an antihypertensive agent of choice in patients with multiple cardiovascular risk factors. Considering the 1-year recurrent rate was not different between the two groups, this protective effect of RAAS inhibitors mainly on the cardiovascular system may have also affected our results in the long term.

Since the surgeries for all types of cancers were included in the present analysis, we conducted the subgroup analysis according to cancer types. But we did not find the relationship between the use of RAAS inhibitors and the reduction of 5-year mortality in surgeries for specific cancer types except lung cancer. Therefore, it is hard to conclude the RAAS inhibitor's effect on survival after specific cancer surgeries in this study. However, our result of declining 5-year mortality in the RAAS inhibitor group after lung cancer surgery is consistent with the previous studies in which lung cancer prognosis was associated with RAAS inhibitors, especially in non-small cell lung cancer (NSCLC)^23^. Also, there is an interaction between RAAS inhibitors and epidermal growth factor receptor (EGFR) inhibitor using the crosstalk between AT1R and EGFR, the first-line treatment target for NSCLC^24,25^. Therefore, RAAS inhibitors may delay EGFR inhibitor resistance, theoretically. The heterogeneity of association between the types of surgery may also be related to the fact that power of analysis depends on number of events, so some types of surgery might have not achieved statistical significance due to low mortality.

The following limitations should be considered when interpreting the results of this study. First, as a single-center, observational study, a residual confounding factor-related bias may have persisted, despite our rigorous statistical adjustment. Unmeasured confounding variables may have affected the results even in the propensity-score matched population. Second, due to retrospective properties, it was impossible to include the blood pressure changes from discharge to follow-up in this study. So, we cannot rule out the possibility that uncontrolled hypertension or inadvertent hypotension due to RAAS inhibitors might have contributed to the death. Third, we did not consider the types and doses of RAAS inhibitors. Moreover, considering most of the patients were prescribed angiotensin II receptor blockers, our results may differ for angiotensin-converting-enzyme inhibitors. As a result, the potential effect of different and interrupted use of RAAS inhibitors and dose–response relationships could not be ascertained. In addition, our analysis may show different results according to types of surgery. Fourth, prescription from other clinics after the discharge could not be considered. So, some of RAAS inhibitor prescription after discharge might have been misclassified as no RAAS inhibitor group. Finally, our study mainly included Asians, and the findings may not be generalized to other ethnicities. Despite these limitations, we used real-world data and demonstrated that the use of RAAS inhibitors after cancer surgery was associated with reduced mortality and recurrence rate.

## Conclusion

Our study showed that the use of RAAS inhibitor decreased 5-year mortality in hypertensive patients after cancer surgery. However, large-scale, well-designed studies are needed for evaluating the relationship between the use of RAAS inhibitor and the outcomes after cancer surgeries. Based on the present evidence including our data, prescription of RAAS inhibitors as a first-line antihypertensive agent seems to be reasonable in patient who underwent cancer surgery.

## Supplementary Information

Supplementary Tables.

## Abbreviations

ACE: Angiotensin-converting enzyme
ASD: Absolute standardized difference
AT1R: Angiotensin II type 1 receptor
AT2R: Angiotensin II type 2 receptor
CI: Confidence interval
EGFR: Epidermal growth factor receptor
HR: Hazard ratio
IQR: Interquartile range
NSCLC: Non-small cell lung cancer
RAAS: Renin–angiotensin–aldosterone system
SD: Standard deviation
SMC-CanSur: Samsung Medical Center Cancer Surgery
