# Supplementary material for: The use of renin angiotensin aldosterone system inhibitors may be associated with decreased mortality after cancer surgery
Source: Sci Rep. 2022 Apr 27;12:6838. doi: 10.1038/s41598-022-10759-y (PMC9046295; doi:10.1038/s41598-022-10759-y)
Supplement: Supplementary file 1 — Supplementary Tables. [file 41598_2022_10759_MOESM1_ESM.docx]

| **Supplementary Table 1.** Antihypertensive drugs use |  |  |  |  |  |  |  |
| --- | --- | --- | --- | --- | --- | --- | --- |
|  | Entire population | | | | Propensity-score matched population | |  |
|  | No RAAS  inhibitor | RAAS  inhibitor |  |  | No RAAS inhibitor | RAAS  inhibitor |  |
|  | (n = 8374) | (n = 11391) |  |  | (n = 5022) | (n = 5022) |  |
| Preoperative medication |  |  |  |  |  |  |  |
| RAAS inhibitor | 485 (5.8) | 6854 (60.2) |  |  | 485 (9.7) | 485 (9.7) |  |
| ARB | 391 (4.7) | 6545 (57.5) |  |  | 391 (7.8) | 462 (9.2) |  |
| ACEi | 97 (1.2) | 360 (3.2) |  |  | 97 (1.9) | 29 (0.6) |  |
| Duration of RAAS inhibitor use, days | 503 (320-725) | 531 (315-732) |  |  | 522 (325-752) | 460 (302-615) |  |
| Beta blocker | 1672 (20.0) | 1388 (12.2) |  |  | 1051 (20.9) | 341 (6.8) |  |
| Calcium channel blocker | 3807 (45.5) | 3316 (29.1) |  |  | 2319 (46.2) | 824 (16.4) |  |
| Duration of any antihypertensive drug use, days | 721 (502-921) | 636 (458-827) |  |  | 721 (501-918) | 495 (304-728) |  |
| Postoperative medication |  |  |  |  |  |  |  |
| RAAS inhibitor |  | 11391 (100) |  |  |  | 5022 (100) |  |
| ARB |  | 10974 (96.3) |  |  |  | 4889 (97.4) |  |
| ACEi |  | 442 (3.9) |  |  |  | 138 (2.7) |  |
| Duration of RAAS inhibitor use, days |  | 452 (325-568) |  |  |  | 378 (253-526) |  |
| Beta blocker | 1891 (22.6) | 1039 (9.1) |  |  | 1154 (23.0) | 388 (7.7) |  |
| Calcium channel blocker | 7120 (85.0) | 5675 (49.8) |  |  | 4251 (84.6) | 2711 (54.0) |  |
| Duration of any antihypertensive drug use, days | 531 (318-756) | 541 (339-793) |  |  | 538 (321-768) | 420 (238-684) |  |
| Data are presented as n (%) or median (interquartile range) | |  |  |  |  |  |  |
| RAAS, renin-angiotensin-aldosterone system; ARB, Angiotensin II receptor blockers; ACEi, Angiotensin-converting-enzyme inhibitors | | | | | | |  |
|  | | | | | | |  |
|  | | |  |  |  |  |  |

**Supplementary Table 2.** Types of cancer

|  | Entire population | | Propensity-score matched population | |
| --- | --- | --- | --- | --- |
|  | No RAAS inhibitor | RAAS inhibitor | No RAAS inhibitor | RAAS inhibitor |
|  | (n = 8374) | (n = 11391) | (n = 5022) | (n = 5022) |
| Neuroendocrine | 527 (6.3) | 552 (4.8) | 366 (7.3) | 242 (4.8) |
| Lung | 1694 (20.2) | 1729 (15.2) | 1001 (19.9) | 623 (12.4) |
| Head and Neck | 1113 (13.3) | 1337 (11.7) | 658 (13.1) | 524 (10.4) |
| Breast | 737 (8.8) | 1110 (9.7) | 429 (8.5) | 479 (9.5) |
| Stomach | 979 (11.7) | 1616 (14.2) | 612 (12.2) | 859 (17.1) |
| Hepatobiliary | 718 (8.6) | 1048 (9.2) | 456 (9.1) | 369 (7.3) |
| Colorectal | 1227 (14.7) | 1768 (15.5) | 728 (14.5) | 846 (16.8) |
| Urologic | 997 (11.9) | 1700 (14.9) | 554 (11.0) | 868 (17.3) |
| Gynecologic | 256 (3.1) | 357 (3.1) | 144 (2.9) | 148 (2.9) |
| Bone, skin, etc | 126 (1.5) | 174 (1.5) | 74 (1.5) | 64 (1.3) |

**Supplementary Table 3.** Effect of an unmeasured confounder on HR of RAAS inhibitor use for 5-year mortality after cancer surgery in the propensity-score matched analysis

|  |  | OR*_ZY_*_\|_*_X_* | | | | | | |
| --- | --- | --- | --- | --- | --- | --- | --- | --- |
|  |  | 1.5 | | 2 | 2.5 | 3 | 3.5 | 4 |
| OR_zx_ | 0.3 | 0.79 (0.69-0.91) | 0.86 (0.75-0.98) | | 0.92 (0.80-1.05) | 0.97 (0.84-1.11) | 1.01 (0.88-1.51) | 1.03 (0.90-1.18) |
|  | 0.4 | 0.78 (0.68-0.89) | 0.84 (0.73-0.96) | | 0.87 (0.76-0.99) | 0.91 (0.79-1.04) | 0.94 (0.82-1.07) | 0.95 (0.83-1.08) |
|  | 0.5 | 0.77 (0.67-0.87) | 0.80 (0.70-0.92) | | 0.83 (0.73-0.95) | 0.87 (0.76-0.99) | 0.88 (0.77-1.01) | 0.90 (0.79-1.02) |
|  | 0.6 | 0.76 (0.67-0.87) | 0.79 (0.69-0.90) | | 0.80 (0.71-0.92) | 0.81 (0.71-0.93) | 0.84 (0.74-0.96) | 0.85 (0.74-0.97) |
|  | 0.7 | 0.75 (0.66-0.86) | 0.77 (0.67-0.87) | | 0.79 (0.69-0.90) | 0.79 (0.69-0.90) | 0.80 (0.70-0.91) | 0.81 (0.71-0.92) |

Prevalence of unmeasured confounder = 40%

Numbers represent HRs (including 95% CIs).

RAAS, renin-angiotensin-aldosterone system; HR, hazard ratio; OR, odds ratio; X: dichotomous exposure measure, y dichotomous outcome measure, z : potential dichotomous confounder.

ORZX indicates the association (OR) between the unmeasured confounder and RAAS inhibitor use.

ORZY|X indicates the association (OR) between the unmeasured confounder and 5-year mortality after cancer surgery.
